# A Universal Nano‐capillary Based Method of Catalyst Immobilization for Liquid‐Cell Transmission Electron Microscopy

**DOI:** 10.1002/anie.201916419

**Published:** 2020-03-02

**Authors:** Tsvetan Tarnev, Steffen Cychy, Corina Andronescu, Martin Muhler, Wolfgang Schuhmann, Yen‐Ting Chen

**Affiliations:** ^1^ Analytical Chemistry—Center for Electrochemical Sciences (CES) Faculty for Chemistry and Biochemistry Ruhr University Bochum 44801 Bochum Germany; ^2^ Industrial Chemistry Faculty of Chemistry and Biochemistry Ruhr University Bochum 44801 Bochum Germany; ^3^ Chemical Technology III Faculty of Chemistry and CENIDE Center for Nanointegration University Duisburg Essen Carl-Benz-Strasse 199 47057 Duisburg Germany; ^4^ Center for Solvation Science (ZEMOS) Ruhr University Bochum 44801 Bochum Germany

**Keywords:** electrocatalysis, ethanol oxidation, liquid-cell TEM, nano electrochemistry, nickel boride

## Abstract

A universal nano‐capillary based method for sample deposition on the silicon nitride membrane of liquid‐cell transmission electron microscopy (LCTEM) chips is demonstrated. It is applicable to all substances which can be dispersed in a solvent and are suitable for drop casting, including catalysts, biological samples, and polymers. Most importantly, this method overcomes limitations concerning sample immobilization due to the fragility of the ultra‐thin silicon nitride membrane required for electron transmission. Thus, a straightforward way is presented to widen the research area of LCTEM to encompass any sample which can be externally deposited beforehand. Using this method, Ni_*x*_B nanoparticles are deposited on the μm‐scale working electrode of the LCTEM chip and in situ observation of single catalyst particles during ethanol oxidation is for the first time successfully monitored by means of TEM movies.

## Graphical abstract

**A novel universal method** of single catalyst nanoparticle immobilization on the working electrode without damaging the fragile membrane allows single Ni_*x*_B nanoparticle catalysts to be monitored in situ during ethanol oxidation by means of TEM movies.

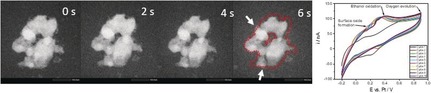

The use of liquid‐cell transmission electron microscopy^1^ (LCTEM) has been reported providing the basis of atomically resolved observations in liquid, and with various applications including materials synthesis/processing/corrosion,^2^ mineralogy and geochemistry,^3^ batteries,^4^ polymers,^5^ catalytic reactions, and biology.^6^ It allows in situ microscopy in the liquid phase with both high spatial and temporal resolution.^7^ To separate the liquid from the TEM vacuum, the sample is encapsulated between two thin (<50 nm) Si_3_N_4_ membranes (upper and lower) to form a liquid film thin enough to allow electrons to penetrate. Si_3_N_4_ membranes are most common in LCTEM owing to their mechanical and chemical robustness as well as the highly controlled fabrication and assembly. Resolution in TEM can be improved by reducing the sample thickness, and hence graphene is considered as membrane material.^8^ The very small thickness and the fragility of the membrane renders sample preparation very challenging. This may be the major reason, why as compared to other in situ TEM technologies, such as gas‐phase or in situ heating, LCTEM is much less reported in spite of its large demand for many research fields including electrocatalysis. One of the challenges is the difficulty of sample preparation since for applications in electrochemistry the sample, that is, the catalyst material, has to be immobilized exclusively on the working electrode. However, because of its small size and the fragility of the underlying Si_3_N_4_ membrane, techniques commonly used for the modification of macroscopic electrodes, such as drop‐, dip‐coating, or ink‐jet printing, cannot be used. To maintain the integrity of the Si_3_N_4_ membrane it is crucial to resist the pressure difference between inside and outside of the liquid cell. The microscopic dimensions of the three‐electrode compartment pose another challenge because the distance between the three electrodes can be as small as 10 μm, which necessitates a high‐resolution deposition technique to avoid electrical shortcut between the electrodes with the deposited sample itself. Hence, LCTEM examples are mostly limited to samples suspended in the liquid phase or suspensions containing precursors from which the investigated material is deposited. The deposition of a small number or even single particles remains as a big challenge. Ortiz Peña^9^ et al. used a silicon mask with an opening of the same size as the Si_3_N_4_ window of tens of μm to allow the whole window to be covered with many particles after drop casting. Yang^10^ et al. applied a defined potential to the working electrode to attract particles from the suspension and immobilize them on the working electrode surface.^11^ The whole working electrode was randomly covered by the sample and a gradient of the sample amount from the tip to the root of the electrode has been observed. A focused ion beam was proposed for bulk samples,^12^ however, protection of the membrane from the high‐energy ion beam is difficult. Deposition of small amounts of particles from a solution can include inkjet methods with droplet diameters in the range of tens of micrometers, which is too large for LCTEM chip modification.^13^ Atomic force microscopy (AFM) derived methods, such as dip pen nanolithography (DPN) are possible alternatives,^14^ capable of producing features on surfaces with dimensions in the nanometer range. However, the direct contact between the solid tip and the fragile glassy carbon electrode/Si_3_N_4_ membrane can create damage. Therefore, it is crucial to find a universal and easy way to deposit samples exclusively on top of the small and fragile membrane/electrode on the LCTEM chip.

Ideally, a low‐cost, fast and easy to use method for liquid deposition is required and we are suggesting a scanning electrochemical cell microscopy (SECCM)^15^ derived approach using a pulled glass capillary filled with a suspension of the catalyst material for precise catalyst deposition. SECCM has previously been used to study nanoparticles contained in a capillary via detection of impacts and landing events^16^ or particles that were already immobilized on a surface.^17^ Potential‐controlled deposition from a capillary has also been reported.^18^, ^19^, ^20^, ^21^ The capillary approaches the chip surface in air, and the approach is automatically stopped when the droplet at the end of the capillary makes contact with the surface using a fast‐feedback recording of either the current flowing between the surface and the electrode inserted from the back into the capillary or using vibration of the SECCM theta capillary and measuring the current or current magnitude between the two capillaries across the droplet.

Upon contact of the surface with the droplet and wetting of the sample surface, particles from the suspension are deposited at the droplet landing site and upon retraction of the capillary a small droplet remains at the surface containing the material to be deposited. We employed a SECCM‐based system to deliver small amounts of a catalyst suspension on a scale of femtoliters to the working electrode of a LCTEM chip (Scheme 1).

**Scheme 1:**
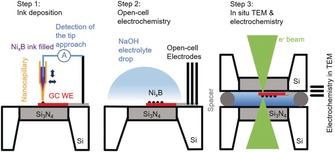
In step 1, a Ni_*x*_B ink filled capillary is approached to the glassy carbon working electrode of a LCTEM chip using a SECCM system until a fast feedback loop stops the approach upon contact of the liquid meniscus at the end of the capillary with the chip surface thereby preventing membrane damage. The Ni_*x*_B ink is transferred to the electrode surface. Step 2, open‐cell electrochemistry is performed to verify successful deposition of the catalyst Ni_*x*_B by conducting electrochemical experiments in an electrolyte droplet covering the three‐electrode chip. Step 3 refers to the fully assembled liquid TEM chip as it is mounted within the TEM.

Ni_*x*_B nanoparticles were used to investigate ethanol oxidation. Ni_*x*_B was reported as highly active and stable electrocatalyst for the oxygen evolution reaction (OER) in alkaline media.^22^ Operando XAFS measurements indicate the formation of an NiO_*x*_H shell surrounding a Ni_*x*_B core under oxygen evolution conditions.^23^ Moreover, an OER catalyst based on Ni_3_B cores with Ni(BO_2_)_2_ shells^24^ and the suitability of Ni_*x*_B tubes for alcohol oxidation reactions was described.^25^ Typically, pre‐ and post‐electrochemistry TEM analysis is employed to evaluate changes in catalyst morphology and structure at the applied high oxidation potentials to invoke the OER. The in situ TEM observation of structural catalyst modifications during electrocatalysis, despite being of high importance for the interpretation of catalyst stability, has not yet been reported.

Owing to the fact that the amount of possible applicable catalysts and the volume of electrolyte for in situ liquid TEM are highly limited we present a straight‐forward drop‐casting technique of an electrocatalyst ink onto the micrometer sized working electrode (WE) of a LCTEM chip.

The steps of the proposed experimental sequence are depicted in Scheme 1. A nanocapillary was prepared by pulling quartz tubes with a laser puller controlling its size in a range of several tens of nanometers to several tens of micrometers by tuning the pulling parameters. The orifice of the pulled capillary largely determines the later amount of deposited catalyst ink on the WE surface. The pulled nanocapillary was filled with a suspension containing Ni_*x*_B nanoparticles in ethanol. A Pt wire was inserted into the capillary from the back and connected to a current amplifier. A conductive tape was used to connect the fragile working electrode to the current amplifier via the corresponding Pt contact pad at the edge of the chip. During the approach of the capillary to the chip surface, when the tiny electrolyte droplet protruding from the orifice of the capillary touches the glassy carbon electrode on the Si_3_N_4_ membrane, the electric circuit between the WE and the Pt electrode inside the capillary is closed and a current signal is detected by the current amplifier. This signal was used as the feedback criterion to automatically stop the approach before the glass body of the capillary contacts the WE, thereby protecting both the Si_3_N_4_ membrane and the glassy carbon electrode from damage. As a result of the capillary force created upon touching, ethanol/Ni_*x*_B particles are delivered from the capillary to the WE surface. After retraction of the capillary, the ink left on the electrode dries in seconds, and hence the Ni_*x*_B particles are immobilized by van der Waals forces between the particles and the glassy carbon surface. The approach to the WE can be repeated after retracting the capillary and displacing it along the WE.

After sample deposition, a 0.5 μL electrolyte droplet containing 0.15 m NaOH and 2 m EtOH was dropped on top of the active area of the chip covering all three electrodes (Step 2 in Scheme 1). The electrodes were connected to the current amplifier with flexible pins to evaluate the ethanol oxidation reaction by means of cyclic voltammetry. Afterwards, the electrolyte was removed with water and ethanol to prevent formation of NaOH crystals. The third step is to assemble the liquid chip by placing another chip with a second Si_3_N_4_ membrane and spacers on top of the modified LCTEM chip to form the sealed liquid cell on the TEM holder.

Sample deposition and open‐cell electrochemical measurements are shown in Figure 1. Prior to the open‐cell measurements the open circuit potential of the Pt reference electrode on the chip was measured versus a Ag/AgCl/3 m KCl reference electrode to record the potential difference for later calibration during the LCTEM measurements. As shown in Figure 1 e, the CVs recorded in open‐cell arrangement show oxidation peaks at around 0.7 V versus Pt (corresponding to ca. 0.515 V vs. Ag/AgCl/3 m KCl), which are attributed to the formation of the NiOOH on the catalyst particle surface immediately followed by ethanol oxidation.^26^ The strong current increase with increasing potentials is caused by the OER. In the cathodic scan a peak attributed to the reduction of the surface NiOOH can be seen at around 0.52 V vs. Pt (ca. 0.335 V vs. Ag/AgCl/3 m KCl).

**Figure 1:**
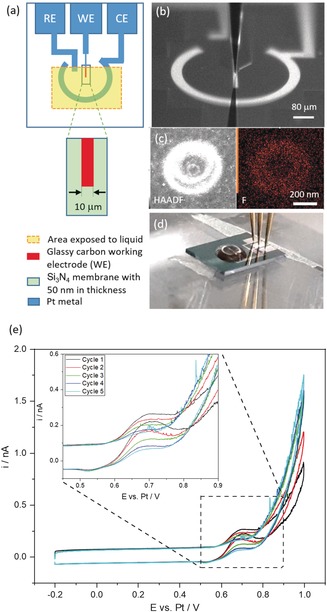
a) LCTEM chip for in situ liquid TEM measurements. The small and thin glassy carbon WE is located on top of a 50 nm thin Si_3_N_4_ membrane which is fragile and breaks upon any mechanical contact. b) Optical microscopy image showing the deposition process. c) Ex‐situ EDS map after the deposition by means of a 500 nm tip. The catalyst modified electrode surface is of similar size as the capillary opening. The fluorine map shows the residual Nafion added to the ink. d) Chip connected with flexible electric contacts to perform open‐cell electrochemistry. e) Cyclic voltammograms recorded from a Ni_*x*_B deposition using a 100 μm tip in the open cell electrochemistry configuration.

The intensity of this peak is very low compared to the background current due to the small surface area of the catalyst particles compared to the area of the entire glassy carbon WE. The small size of this peak relative to the oxidation peak, however, is an indication that the ethanol oxidation reaction is taking place, as in the absence of ethanol the ratio of the peaks is expected to be 1:1. The CV confirms that there is no damage of the Si_3_N_4_ membrane after the deposition of Ni_*x*_B and the circuit is well‐connected.

The successful deposition of Ni_*x*_B particles by means of 7 consecutive SECCM‐type capillary approaches to the GC WE is shown as optical microscope images in Figure 2 a in 200‐fold and Figure 2 b in 1000‐fold magnification. Although the transparent thin GC WE on the Si_3_N_4_ membrane is responsible for a low image contrast, clusters of Ni_*x*_B particles and their positions can be observed. The successful deposition of particles can be further verified by means of in situ STEM‐EDX mapping in the LCTEM, as shown in Figure 2 c. To investigate the applicability of the proposed catalyst immobilization 10 CVs in the potential range between −0.2 to 0.9 V vs. Pt are conducted with a scan rate of 0.5 V s^−1^. In the forward scan the formation of surface oxides of Ni is observed at around 0.2 V. Ethanol oxidation is seen as a broad peak at 0.4 V. Subsequent to the ethanol oxidation peak, the current decreases because of diffusion limitation and possibly surface deactivation before the OER becomes apparent (Figure 2 d).

**Figure 2:**
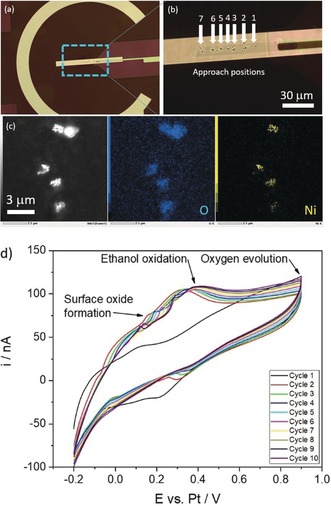
a) and b) show successful Ni_*x*_B depositions obtained by 7 consecutive capillary approaches to the WE, additionally confirmed by the in situ EDS map in liquid as shown in (c). In situ cyclic voltammetry is performed for 10 cycles from −0.2 to 0.9 V versus Pt at a scan rate of 500 mV s^−1^ during electrolyte flow through the liquid cell at 60 μL h^−1^.

To further investigate the shrinkage of the Ni_*x*_B particles during electrocatalysis a chronoamperometric measurement at 0.8 V was recorded while recording a TEM movie with images in 2 s steps (Figure 3 a). Partial dissolution of the particles is observed during the course of 6 s. After ethanol oxidation reaction (EOR) a substantial shrinkage of the catalyst particle can be observed (Figure 3 c) and further verified by in situ energy‐dispersive X‐ray spectroscopy (EDS) in liquid (Figure 3 d).

**Figure 3:**
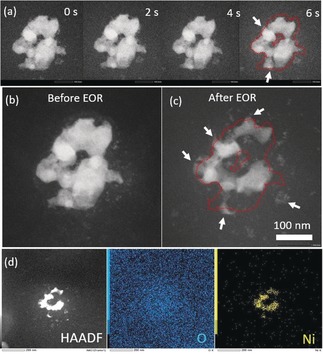
a) In situ LCTEM imaging of the morphological change of a Ni_*x*_B particle during a chronoamperometric experiment at 800 mV versus Pt for 6 s (first image recorded at the start of the experiment). The STEM‐HAADF image series is extracted from a corresponding movie (Supporting Information Movie S1) recorded using LCTEM. The shape of particle at 0 s is marked as red dashed line in the image after 6 s (b) and (c). The same particle before and after 10 CV cycles exhibited large morphological changes. In addition, Ni is nucleated and grown at particle edges as well as in the vicinity of them, as indicated by arrows in (c). d) In situ EDS mapping showing traces of Ni and O confirming the elemental distribution after electrocatalysis. The newly formed small particles are composed of Ni.

Dissolution of single Ni_*x*_B particles, or single electrocatalyst particles generally, was not frequently reported until now because electrocatalyst dissolution remains unnoticed in macroscopic electrochemical measurements. Geiger et al.^27^ reported possible dissolution of tin‐based electrocatalysts facilitated by anodic potentials. In the case of Ni_*x*_B, anodically formed nickel oxides and oxyhydroxides constitute the electrochemically active phase. Dissolution of the electrocatalyst may be facilitated by the consumption of formed oxides during alcohol oxidation.^28^ Another competing driving force for Ni dissolution is the reduction of Ni by the electron‐beam, which results in the nucleation and growth of Ni particles due to the high concentration of Ni in the solution. The growth of Ni particles was observed causing the expansion of the particle at the edge as well as nucleation of small particles nearby as indicated by white arrows in Figure 3 a and 3 c.

In conclusion, using a nanocapillary, deposition of catalyst particles from inks can be achieved on the fragile GC/Si_3_N_4_ membrane without direct contact between the glass walls of the capillary and the chip surface. Once established, the method is clean, easy to use, and easy to tune as the amount of deposited sample can be controlled by selecting capillaries with different size. Owing to the small amount of deposited liquid compared to the whole volume of the capillary and the ability to perform approaches stably and reproducibly, multiple approaches can be performed with the same capillary. The first successful in situ observation of single catalyst particles during ethanol oxidation has been achieved with this method.

## Supporting information

As a service to our authors and readers, this journal provides supporting information supplied by the authors. Such materials are peer reviewed and may be re‐organized for online delivery, but are not copy‐edited or typeset. Technical support issues arising from supporting information (other than missing files) should be addressed to the authors.

Supplementary

Supplementary

Supplementary

Supplementary
