# Supplementary material for: A Universal Nano‐capillary Based Method of Catalyst Immobilization for Liquid‐Cell Transmission Electron Microscopy
Source: Angew Chem Int Ed Engl. 2020 Mar 2;59(14):5586–90. doi: 10.1002/anie.201916419 (PMC7155139; doi:10.1002/anie.201916419)
Supplement: Supplementary file 1 — Supplementary [file ANIE-59-5586-s001.pdf]

Angewandte Chemie
Eine Zeitschrift der Gesellschaft Deutscher Chemiker

Supporting Information

A Universal Nano-capillary Based Method of Catalyst Immobilization for Liquid-Cell Transmission Electron Microscopy

Tsvetan Tarnev, Steffen Cychy, Corina Andronescu, Martin Muhler, Wolfgang Schuhmann, and Yen-Ting Chen**

anie_201916419_sm_miscellaneous_information.pdf
anie_201916419_sm_S1.avi
anie_201916419_sm_S2.avi
anie_201916419_sm_S3.avi

Supporting Information

Experimental Section

LCTEM. Transmission electron microscopy was carried out using a JEOL JEM-2800 working with a Schottky field emission gun (FEG) at 200 kV. It is equipped with Gatan Oneview-IS CCD camera working with 4k x 4k pixels at 25 fps for in situ movie recording. The elemental EDS mapping was implemented with dual EDS SDD detectors of solid angles of 0.95 srad. The STEM-HAADF images were taken at the detection angle of 70-200 mrad, with simultaneously detected bright field and secondary electron images. LCTEM was performed using the liquid E-chip system and a Protochips Poseidon 210 holder with simultaneously flowing electrolyte at 60 μl/h through the active region. The double etched EDS-enhanced chip design and small liquid window were used for high resolution EDS mapping in the liquid.

Ni_xB synthesis. NixB was prepared by reducing $NiCl_2$ with $NaBH_4$. 20 mL 0.5 M $NiCl_2$ aqueous solution were deaerated by Ar saturation in a round-bottomed Schlenk flask and the solution was cooled to 0 °C in an ice bath. An equally deaerated 20 mL 1 M $NaBH_4$ solution was added dropwise via a syringe under constant Ar feed. The black precipitate was collected by filtration, washed with 1 L distilled water and subsequently 100 mL ethanol. The washed filtrate was annealed for 2 h at 500 °C in 20 mL min^{-1} N_2 atmosphere with a heating rate of 10 K min^{-1}.

Nanocapillary and SECCM setup. Nanocapillaries were prepared with a laser-based micropipette puller (Sutter P-2000) using quartz capillaries of 1.2 and 0.9 mm outer and inner diameter, respectively. The pulled capillaries were filled with the Catalyst nanoparticle containing ink using a MicroFil needle (MF34G-5, World Precision Instruments). A Pt wire was inserted into the capillary and the ink-filled capillary was mounted on a specifically designed capillary holder. The working electrode on the LCTEM chip was connected via its Pt contact pad using a Cu tape and the chip was positioned on a x,y,z-piezocube (P-611.3S nano cube, PI) controlled by an analogue amplifier (E-664, PI). The piezo cube is fixed on positioning system consisting of three stepper motors (Owis) controlled with a LStep PCIe (Lang) controller. The stepper motors are used to preposition the capillary at around 10 µm above the working electrode of the chip controlled optically with the aid of a video microscope camera (DMK 21AU04, The Imaging Source). Automatic approach is started with an approach speed of 100 nm/s, while applying a voltage difference of 100 mV between the Pt wire inside the capillary and the working electrode of the chip and monitoring the current with a current amplifier (ELC-03XS, NPI). Initially only noise is measured as the electric circuit is not closed, however, immediately upon contact of the drop hanging from the opening of the capillary with the surface, a distinct increase in the measured signal is observed, which can be used to stop the approach in an automatic feedback loop. The stop criterion was usually set to the detection of a current higher than 20 pA. The capillary is then retracted manually and moved to another position above the chip for the next approach in order to increase the catalyst loading. The set-up is built on a vibration damping table (RS 2000, Newport) with S-2000 stabilizers (Newport) in a Faraday cage with thermal isolation panels (Vaku-Isotherm). The piezo cube, stepper motors and current amplifier are controlled via a FPGA card (PCIe-7852R, National Instruments) with a LabVIEW software adapted on the basis of the program obtained from the Unwin group, University of Warwick (WEC-SPM).

Determination of the reference voltage by open circuit potential measurements. The open circuit potential of the reference electrode on the chip was measured vs a conventional Ag/AgCl/3 M KCl in a solution containing 0.15 M NaOH and 2 M ethanol using a Jaissle potentiostat/galvanostat (1002 PC.T). A value of -185 mV was obtained.

Open-cell electrochemical measurements. After successful deposition of catalyst nanoparticles, the Pt contact pads of the chip for the working and counter electrodes were connected with flexible pins to the current amplifier. A 0.5 µL of a solution containing 0.15 M NaOH and 2 M EtOH was dropped on the chip covering all electrodes but not the connection pads. CVs were recorded from -0.2 to 1 V vs Pt with a scan rate of 50 mV/s.

This supporting information contains 3 movies recorded during the in situ observation with LCTEM.

S1: Movie of In situ TEM with normal speed. The single NiB particle was not affected by the electron beam illumination at the beginning. At around 19th second, the voltage of chronoamperometry was applied to the working electrode and a significant morphological change could be observed.

S2. The observation of a group of particles shows the successful deposition on the glassy carbon electrode with the flowing electrolyte.

S3. After the CV the morphology of single particle is changed.
